# Persistent Calyxes in Postbloom Fruit Drop: A Microscopy and Microanalysis Perspective

**DOI:** 10.3390/pathogens9040251

**Published:** 2020-03-28

**Authors:** João Paulo Rodrigues Marques, Marcel Bellato Spósito, Lilian Amorim, Gabriel Sgarbiero Montanha, Geraldo José Silva Junior, Hudson Wallace Pereira de Carvalho, Beatriz Appezzato-da-Glória

**Affiliations:** 1 Center of Nuclear Energy in Agriculture, University of São Paulo, Piracicaba 13400-970, Brazil; joaoanatomia@gmail.com (J.P.R.M.); gabriel.montanha@usp.br (G.S.M.); 2 “Luiz de Queiroz” College of Agriculture, University of São Paulo, Piracicaba 13418-900, Brazil; mbsposito@usp.br (M.B.S.); lilian.amorim@usp.br (L.A.); 3 The Brazilian Fund for Citrus Protection, Araraquara 14807-040, Brazil; geraldo.silva@fundecitrus.com.br

**Keywords:** calcium oxalate crystals, citrus, fungal disease, starch, µ-XRF

## Abstract

Citrus postbloom fruit drop, caused by *Colletotrichum* spp., is an important disease in the Americas. The pathogen infects citrus flowers, produces orange-brown lesions on petals, and may cause the abscission of young fruit. In diseased flowers, the calyxes remain attached to the peduncle after the young fruit drop. No anatomical and microanalysis studies have been conducted to determine whether calyx tissues can be infected by *Colletotrichum* spp. and why calyxes remain attached to the peduncle. Based on light microscopy, we demonstrate that the ovary abscission zone exhibits a separation region composed of layers of thickened lignified walled cells, indicating that abscission involves the disruption of cell walls. The first layers of the protective zone (PZ) are composed of densely packed cells with suberized walls produced by the wound meristem. Beneath the PZ, there is a compact mass of small cells that accumulate starch grains. X-ray fluorescence microanalysis (µ-XRF) confirmed the increased accumulation of calcium in the receptacle of the persistent calyxes compared to non-inoculated citrus flowers. Moreover, the peduncle pith and the receptacle exhibit hypertrophied cells with thick walls that may be related to calyx retention. Fungal structures are not observed inside the persistent calyx tissues.

## 1. Introduction

Postbloom fruit drop (PFD) is an important citrus disease caused by *Colletotrichum* spp. The first reports indicated *Colletotrichum acutatum* and *C. gloesporioides* as causal agents [1], and in 2015, *C. abscissum* was described as a PFD pathogen belonging to the *C. acutatum* complex [2,3,4,5]. PFD is responsible for causing serious economic losses to citrus growers, and it is considered a limiting factor for citrus production in countries of Central America and Brazil [6]. The presence of PFD in the orchard can lead to yield losses that can reach up to 93% [7]. Recently, the disease assumed great prominence in the state of São Paulo, the largest Brazilian sweet orange producer [8,9]. 

*Colletotrichum* spp. are responsible for causing orange lesions on petals, which can coalesce and compromise the entire surface and colonize all its tissues [10,11,12]. The stigma may also present lesions when infected, but these lesions are necrotic and localized since the fungus does not colonize the stigma tissues [13]. After the period of the colonization of petal tissues, premature fall of the ovary occurs, as does the production of persistent calyxes or “starlets”. Persistent calyxes are diagnostic for the disease [14]. In contrast to natural young fruit drop, fruitlet abscission caused by *Colletotrichum* spp. occurs between the calyxes and young fruit [15]. The calyxes continue to enlarge after fruitlet abscission and remain firmly attached to the branches for 18 months or more [5]. The presence of this structure has been discussed as a source of inocula of *Colletotrichum* spp. in the orchard [15], which could contribute to pathogen survival [9].

The fruitlet abscission caused by PFD occurs at the base of the ovary, in contrast to the natural fruit abscission of the young fruit between the peduncle and the branch [16,17]. The infection of *Colletotrichum* spp. in citrus flowers leads to changes in the hormonal balance and consequent early abscission of the ovary [16]. Hormonal changes, especially in the differential expression of genes related to the production and regulation of indolylacetic acid (IAA), ethylene, and jasmonic acid, have been found in PFD-infected tissues [18].

Lahey et al. [19] added that the high levels of IAA detected (increased by 140 times) may contribute to the increase in calyx thickness. The use of auxin transport inhibitors such as TIBA (2,3,5-triiodobenzoic acid) and 2,4-D promotes higher retention of fruits after fungal infection compared to flowers treated only with water [16]. This information reinforces the hypothesis that auxin plays an important role in premature fruit drop. The application of synthetic gibberellins also favours greater retention of fruits from infected flowers [16].

The present work aimed to describe the anatomical and histochemical structure of citrus flowers with and without PFD symptoms and to demonstrate the process of calyx retention. Additionally, the presence of the pathogen and the distribution of calcium within a persistent calyx were assessed.

## 2. Results

### 2.1. Anatomy of Healthy Receptacle and Peduncle

**Figure 1:**
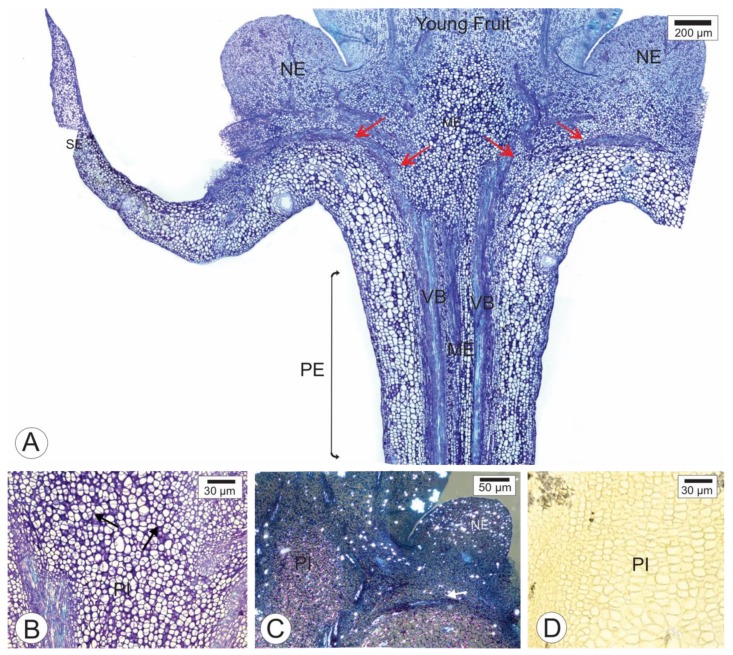
Longitudinal sections of the healthy floral receptacle and peduncle of *Citrus sinensis* cv. Valencia. (**A**–**C**). Toluidine blue staining method. (**A**). Overview of the peduncle, sepals, nectary, and base of the young fruit attached to the receptacle. The arrows indicate the ramifications of the vascular bundles in the receptacle. (**B**). Detail of the pith of the receptacle, where dividing cells are noticeable (arrows). (**C**). Analysis under polarized light. Note the presence of crystals in the nectary and scattered in the receptacle. (**D**). Negative reaction for zinc chloride iodine reaction in receptacle pith cells. PE—floral peduncle; PI—pith; NE—nectary; SE—sepals.

In longitudinal sections of healthy floral receptacles and peduncles of *Citrus sinensis* ‘Valencia’ (Figure 1A), the sepals, nectary, and base of the young fruit were observed. In this stage, the petals and androecium had fallen. The distribution of the vascular traces in the different whorls was observed in the receptacle region. The parenchyma of the receptacle and the peduncle presented numerous cell divisions (Figure 1B). Under polarized light, we observed numerous calcium oxalate crystals sparsely distributed in the nectary and the receptacle pith (Figure 1C). Histochemical tests with zinc iodine chloride revealed the absence of starch in the receptacle pith cells (Figure 1D).

### 2.2. Anatomy of the Persistent Calyx

**Figure 2:**
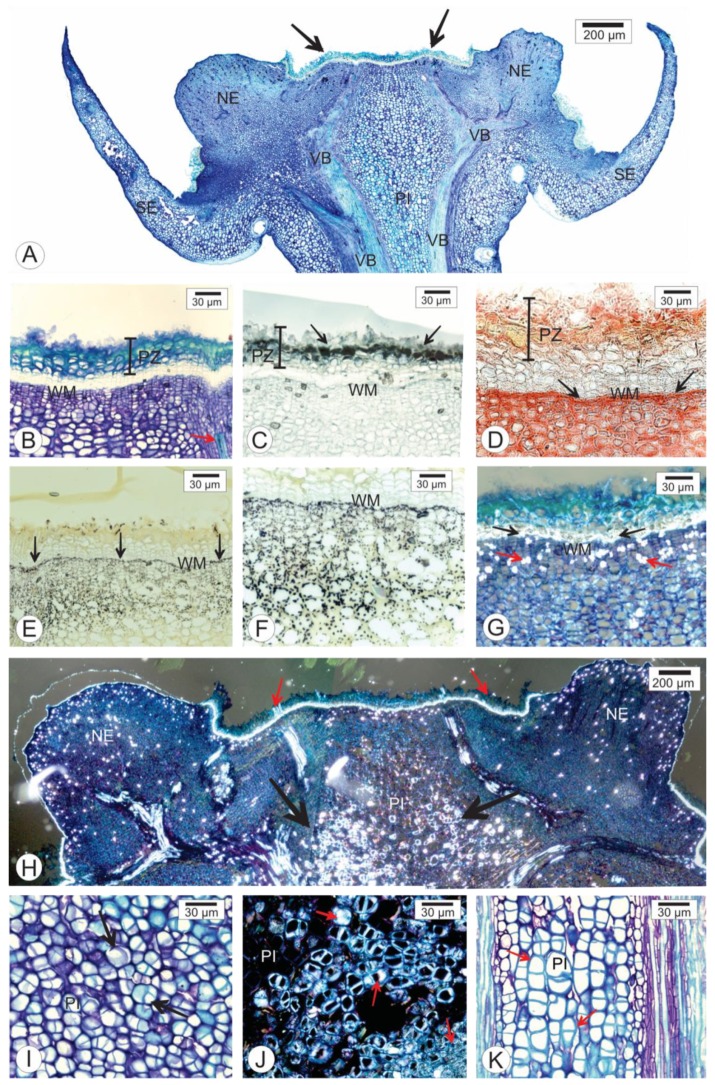
Longitudinal sections of the receptacle of *Citrus sinensis* cv. Valencia showing symptoms of persistent calyx caused by *Colletotrichum acutatum* infection (**A**). Overview of the injured receptacle. Note the wound meristem that underlies the area where the ovary was previously located (arrows). (**B**–**D**). Details of the protective layer (PL). (**C**). Lipid compounds revealed by Sudan black B in the cell layers produced by the meristem (arrows). (**D**). Xylidine Ponceau test indicating the accumulation of protein compounds (arrows) in meristematic cells. (**E**,**F**). Positive reaction for iodinated zinc chloride, indicating that starch accumulates beneath the entire length of the wound meristem (arrows). (**G**,**H**,**J**). Analysis of sections under polarized light. (**G**). Deposition of crystals between cells (red arrows). (**H**). Cellulose thickening of the pith cell walls (larger arrows). (**I**). Detail of pith with cells that have undergone hypertroph and have thick walls (arrows). (**J**). Note the presence of crystals (arrows). (**K**). Hypertroph pith cells with thick walls (arrows). NE—nectary; PI—pith; WM—wound meristem; VB—vascular bundle.

**Figure 3:**
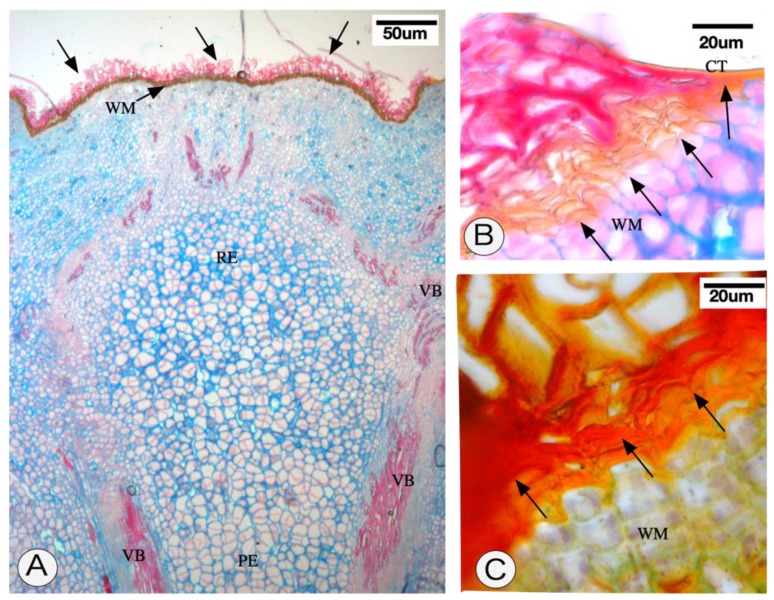
Longitudinal sections of the peduncle and receptacle of persistent calyx in *Citrus sinensis* cv. Valencia caused by *Colletotrichum acutatum* infection. (**A**–**B**). Staining method using Alcian blue safranin O, and Sudan IV. It is possible to observe lignified cell walls (arrows in (**A**)) and cells with suberized walls (arrows in (**B**)) above the wound meristem. (**C**). Details of the wound meristem and the cells with thick walls stained with Sudan IV. Strong staining was observed in the cells (arrows). CT— cuticle; PE—peduncle; WM—wound meristem; VB—vascular bundle.

**Figure 4:**
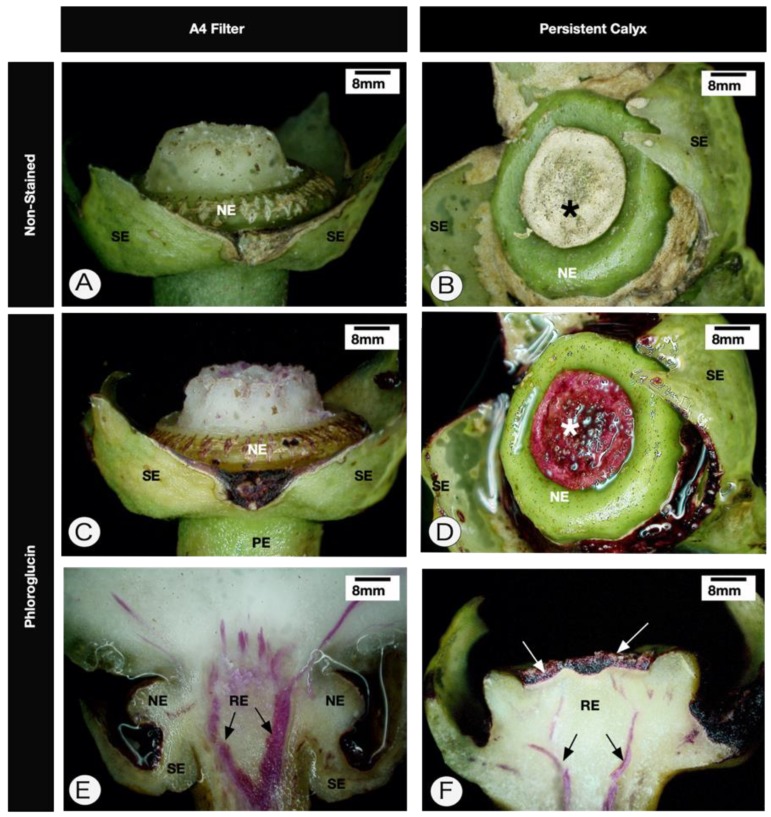
Digital microscopy analysis of *Citrus sinensis* cv. Valencia healthy calyxes and persistent calyxes caused by *Colletotrichum acutatum* infection. (**A**,**C**). The young fruit was removed at the top of the receptacle. (**A**,**B**). Nonstained tissues. (**C**–**F**). Phloroglucin-stained tissues (pink). (**E**,**F**) Longitudinal section. Note that the region where the ovary was inserted has cells with lignified walls (* in **B**,**D**). Black arrows in (**E**,**F**) indicate the vascular bundle. The white arrows in (**F**) indicate the protective layer. NE—nectary; PE—peduncle; RE—receptacle; SE—sepals.

Receptacles with persistent calyx symptoms are larger than receptacles without symptoms. The main characteristics of this structure are greater rigidity of the receptacle and the presence of an abscission zone of ruptured cells (outer ones) and cells with lignified walls (Figure 2, Figure 3A,B and Figure 4), followed by an apical protective zone (Figure 2 and Figure 3). Soon after ovary fall, periclinal divisions of the parenchyma beneath the abscission layer give rise to a wound meristem (Figure 2B and Figure 3). This meristem produces cells with an increased lipid content (Figure 2C) and suberized thick walls (Figure 3B,C) of the protective zone. This lipophilic barrier is continuous to the cuticle (Figure 3B). These characteristics are observed throughout the receptacle, where the ovary or young fruit was once present. The meristematic region presents cells containing protein (Figure 2D) and starch (Figure 2E,F). The deposition of crystals in the protective zone was also observed (Figure 2G). Additionally, cell hypertrophy (Figure 2I) and cellulose thickening of the cell walls occurred in the pith of the floral peduncle (Figure 2H–K). There were no apparent structural changes in the nectary disk or sepals.

We observed the presence of a layer of cells undergoing lignification (Figure 2G and Figure 3A) above those that accumulated compounds of a lipophilic nature (Figure 2C and Figure 3B). Another piece of information regarding the lipophilic region is its continuity with the cuticle region (Figure 3B). In these tissues, the cell wall contained lipophilic compounds (Figure 3C). Altogether, the meristematic region, in addition to the lipophilic and lignified tissues, was referred to as the protective zone.

**Figure 5:**
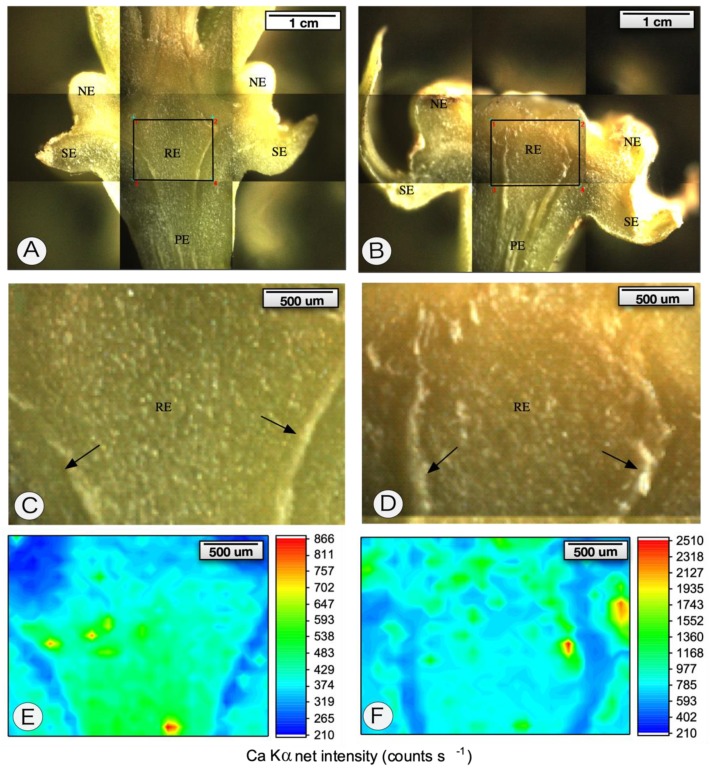
X-ray fluorescence spectroscopy of *Citrus sinensis* cv. Valencia healthy calyxes (**A**,**C**,**E**) and persistent calyxes caused by *Colletotrichum acutatum* infection (**B**,**D**,**F**). The receptacles and peduncles were harvested and longitudinally sectioned. (**A**–**D**). Light microscopy images (63×). (**E**,**F**). X-ray fluorescence microanalysis. Note the high levels of calcium in the persistent calyx. Arrows in (**C**,**D**) indicate the vascular bundle. NE—nectary; PE—peduncle; RE—receptacle; SE—sepals.

Polarized light revealed that the wound meristem presented cells with numerous crystals of calcium oxalate both in intercellular spaces and intracellularly (Figure 2G). We conducted X-ray fluorescence microanalysis (µ-XRF) in order to verify the amount and distribution of calcium in the receptacle of the persistent calyxes. Figure 5 shows that the symptomatic receptacle tissues exhibited higher counts per second of calcium signal compared to the healthy tissues. It was not only the distribution that changed but also the intensity of the signal (Figure 5B,D,F) compared with those in non-inoculated samples (Figure 5A,C,E). 

**Figure 6:**
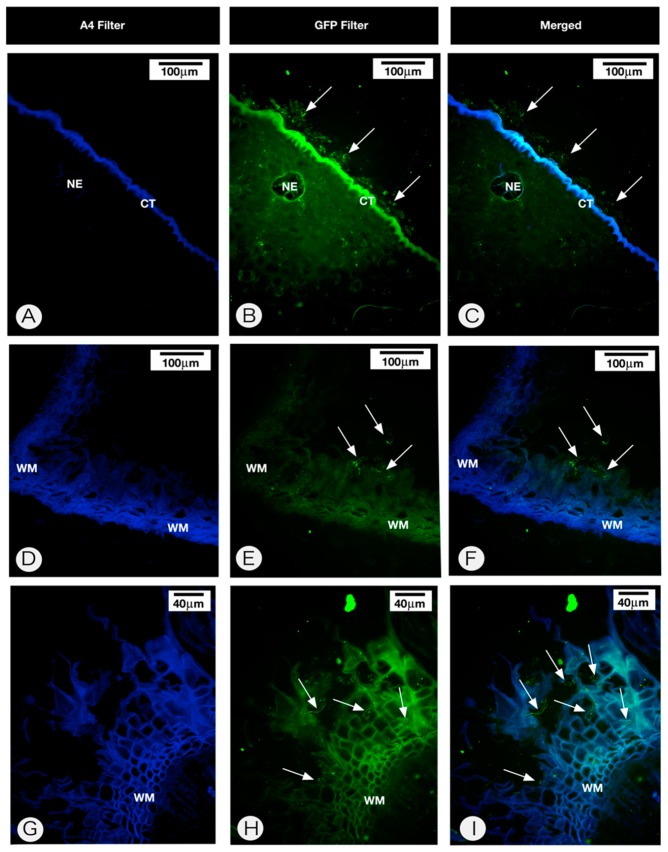
Epifluorescence micrographs of *Citrus sinensis* cv. Valencia persistent calyx caused by *Colletotrichum acutatum* infection. (**A**–**C**). Nectary. Note the thick cuticle and the fungus distributed on the cuticle surface (arrows). (**D**–**I**)—Abscission and protective layers. Note that the fungal hyphae were observed only on the abscission layer surface (arrows in (**E**,**F**)) and between the lignified and suberized cells (arrows in (**H**,**I**)). CT—cuticle; NE—nectary; WM—Wound Meristem.

It should be emphasized that in all the analysed tissues, no hyphae were observed in the internal tissues. To verify this finding, we conducted fungal histolocalization using WGA-Alexa Fluor 488 to detect *C. acutatum* in the persistent calyxes. In all analysed samples, the fungus was only observed in the external tissues. In the nectarous region, the cuticle was thick, and fungal hyphae were only observed on the surface (Figure 6A–C). The fungal hyphae were observed on the abscission layer surface (Figure 6D–F) and between the lignified and suberized cells of the protective layers (Figure 6G–I). No *C. acutatum* hyphae were observed inside receptacle and peduncle tissues.

## 3. Discussion

There are three distinct abscission zones in citrus fruits: I—located between the shoot and the peduncle; II—between the calyx and the fruit; and III—between the fruit and the style [17]. Therefore, our results showed a new abscission zone produced by citrus plants in response to *Colletotrichum acutatum* infection.

When the floral receptacle of the persistent calyx was anatomically investigated, the installation of a protective layer beneath the abscission zone composed of a wound meristem and its products was verified. The installation of such a meristem is a phenomenon observed in lesioned areas in response to biotic or abiotic agents [20,21] and has the function of isolating the internal tissues of the plant from a pathogen or the environment. The cells produced by this meristem accumulate lipophilic compounds and have thick suberized walls. This is a frequent phenomenon in plant tissues in the face of mechanical injury or when the internal tissues are exposed [22]. This lipophilic barrier is continuous with the cuticle, indicating a complex system to prevent the internal tissues from losing water to the atmosphere.

Herein, we showed that the fungus was observable only on the surface of the abscission and protective layers and between their lignified and suberized cells. Citrus species possess a diverse suite of tools designed to overcome pathogen penetration, and similar results associated with the role of the wound meristem were observed in citrus pericarp challenged by *Guignardia citricarpa*, the fungus that promotes black spot disease [23]. Usually, prior to the onset of the abscission process, suberin and lignin contents increase in the cell wall, and starch grains accumulate. Starch and calcium oxalate crystals are ubiquitous responses during abscission layer formation in citrus [17]. Their accumulation was also observed in the present study. Starch likely plays a physiological role as a carbon source for high mitotic activity in the wound meristem.

Microprobe-XRF showed more calcium in persistent calyxes compared to healthy tissues. Herein, it was shown that calcium was mostly homogeneously distributed, even though some spots with high count rates were found (cps). The regions with notable count rates were apparently associated with calcium crystals previously observed with polarized light.

On the other hand, the high levels of calcium in the receptacle tissues indicate that this element may also be associated with the cell walls, linked mainly to low-methyl esterified pectins, forming calcium pectate linkages [24]. Cell walls with high levels of calcium pectate are stiffened [25]. We suggest that the multifaceted role of calcium within the pith of the persistent calyx (receptacle and peduncle) may favour the rigidity of the structure. Moreover, Schneider [26] reported that the thickening of the cell wall of the pith region of the receptacle and the stalk occurs naturally with the development of the fruit. In PFD, after the premature fall of the ovary, we observed the thickening of the cell walls, which stiffened the receptacle and peduncle, favouring their retention in the plant.

Another characteristic is the intense lignification of most external layers of the cells of the protective layer. Lignification is a well-known mechanism of defense of the host against the infection of pathogens [27,28,29]. In the case of PFD, the lignification of the cells may be related to the fall of the ovary and to restraining *C. acutatum* infection due to the absence of fungal hyphae in the analysed tissues.

In summary, we showed herein that the persistent calyx is a complex structure on which the fungus can remain externally attached. No fungal colonization was observed. On the other hand, to the best of our knowledge, this is the first report that shows fungal hyphae on the surface of a persistent calyx, and this structure could be important to the *C. acutatum* lifecycle. Moreover, we described anatomical changes such as cell wall thickening in the receptacle and peduncle pith, which in turn explains why these structures remain attached to the plant for months. Additionally, the µ-XRF analysis showed that these structures accumulated higher contents of calcium than the control plants. This element could be found in the crystal form. No physiological aspects of the nutritional role and starch-based metabolism of persistent calyxes have been described previously, making them a possible target for further studies. Moreover, based on the perspective that persistent calyxes are a source of inocula, we suggest that the removal of the persistent calyxes may be one way to manage the disease in the orchard, avoiding pathogen dispersion.

## 4. Materials and Methods

### 4.1. Plant Material

Four-year-old sweet orange plants (*Citrus sinensis* (L.) Osbeck cv. Valencia) were grown in pots in greenhouses at FUNDECITRUS (The Citriculture Defense Fund), located in Araraquara, Brazil (21°48′27” S, 48°09′54” W, altitude 664 m) and then induced to flower through pruning and water restriction. Healthy and inoculated samples were harvested 30 days after inoculation.

### 4.2. Fungal Inoculation

*Colletotrichum acutatum* inoculation on reproductive branches of ‘Valencia’ sweet orange was performed as described by Marques et al. [12]. For instance, petals were inoculated with the monosporic fungus *C. acutatum* at a concentration of 5 × 105 spores mL^−1^ with the aid of a spray bottle. After spraying, it was necessary to wait approximately 30 min for the placement of plastic bags and the establishment of the humid chambers. In the control plants, distilled water was used. After 30 days, the ovaries fall, and then the calyx and peduncle are retained. The plants were kept in the greenhouse until the end of the experiment.

### 4.3. Light Microscopy

Three young fruits (2–3 cm) and the 4 persistent calyxes (starlet) were collected, sectioned longitudinally and fixed in Karnovsky’s solution [30] (modified with pH 7.2 phosphate buffer). During fixation, the samples were subjected to vacuum. This step was followed by dehydration in an ethyl series and embedding in plastic resin (Leica Historesin^®^, Heraeus Kulzer, Hanau, Germany). The blocks were sectioned at a 5–7 µm thickness on a Leica RM 2045 rotary microtome. The sections were mounted on glass slides and subsequently stained with toluidine blue [31] for the standard histological analyses. Images were captured with a Leica DC 300F video camera attached to the Leica DMLD microscope (Leica, Germany).

### 4.4. Histochemical Tests

Sudan black B was used to detect lipophilic substances [32], with xylidine Ponceau for total proteins [33]. Tests were also carried out with the iodized zinc chloride reagent to detect starch [34]. After staining, the histological slides were mounted in Entellan^®^ synthetic resin (Merck, Darmstadt, Germany). The sections were analysed under polarized light, and the confirmation of their nature was performed on the basis of the solubility of the crystals in 1% sulfuric acid to check for the presence of calcium oxalate crystals [35]. Images were captured with a Leica DC 300F video camera attached to the Leica DMLD microscope (Leica, Germany). To detect lignin in the tissues, the young fruit and the persistent calyxes were longitudinally sectioned and stained with phloroglucin, according to Ruzin [36]. Thus, the samples were analysed under a 3D Digital Microscope (Hirox, Japan).

### 4.5. Fungal Detection

The protocol established by Marques et al. [37] was used to verify the presence of *C. acutatum* hyphae in petal and calyx tissues. The glass slides were treated with a WGA-Alexa Fluor 488 dilution according to the manufacturer (Life^®^) for 30 min and then washed in water once for 3 min. The glass slides were then mounted in water and analysed under a Leica DM 5500 epifluorescence microscope (Leica, Germany). Two filter sets were used: an A4 filter set at 340–380 nm excitation and 450–490 nm emission wavelengths and a GFP filter set at 475–495 and 514–559 emission wavelengths.

### 4.6. µ-XRF Spectroscopy

Three receptacles of both inoculated and non-inoculated samples were longitudinally sectioned and analysed in vivo through the X-ray fluorescence microscopy technique (µ-XRF, Orbis PC EDAX, USA). This microanalysis was carried out using a Rh X-ray tube at 40 kV and 900 µA, selecting a 25 µm Al filter 30 µm X-ray beam. The X-ray sample spectrum was acquired by a silicon drift detector (SDD). The dead time was less than 5%.

Calcium maps were acquired through a 32 × 25 matrix using 1 s per point. The threshold was calculated using the following equation:

Threshold = 8.485 × (BG/T)^−1/2^ (1)

where BG (cps) is the average of 10 random measurements of the background count rate under the corresponding analyte signal, and T is the acquisition time in seconds.
